# Changes in the Fracture Toughness under Mode II Loading of Low Calcium Fly Ash (LCFA) Concrete Depending on Ages

**DOI:** 10.3390/ma13225241

**Published:** 2020-11-19

**Authors:** Grzegorz Ludwik Golewski

**Affiliations:** Department of Structural Engineering, Faculty of Civil Engineering and Architecture, Lublin University of Technology, Nadbystrzycka 40 str., 20-618 Lublin, Poland; g.golewski@pollub.pl; Tel.: +48-81-538-4394

**Keywords:** concrete composite, low calcium fly ash (LCFA), curing time, compressive strength, fracture toughness, mode II loading, development of crack, failure pattern, pozzolanic reaction, microstructure

## Abstract

This study investigated the influence of the curing time on the fracture toughness of concrete produced with different content of low calcium fly ash (LCFA). During the study, the amounts of 20% and 30% of pozzolanic additive were used. In order to observe the effect of the applied pozzolanic additive on the analyzed concrete properties, the obtained results were compared with the values obtained for the reference concrete. Compressive strength—*f*_cm_ and fracture toughness, by using mode II loading—*K*_IIc_ (shearing), were determined between the 3rd and 365th days of curing. In the course of experiments, changes in the development of cracks in individual series of concrete were also analyzed. In addition, the microstructures of all composites and the nature of macroscopic crack propagation in mature concretes were assessed. It was observed that the greatest increase in fracture toughness at shear was in the case of reference concrete during the first 28 days, whereas, in the case of concretes containing LCFA, in the period of time above 4 weeks. Furthermore, concrete without the LCFA additives were characterized by a brittle fracture. In contrast to it, concretes with LCFA additives are mainly characterized by a quasi-plastic process of failure. Moreover, most of the samples showed a typical pattern of the destruction that occurs as a result of shearing. The presented test results may be helpful in selecting the composition of concrete mixtures containing LCFA to be used in concrete and reinforced concrete structures subjected to shear loads.

## 1. Introduction

Concrete is the oldest artificial material with a history of 9000 years [1]. Due to numerous technical and economic values, the production of this material is about 10 billion tons per year, which significantly exceeds the use in the structural engineering of two other important materials, i.e., steel and wood. On the other hand, the main binder for the production of concrete is ordinary Portland cement (OPC), which for several years, is produced at the level of 4.1 billion tons per year [2].

Unfortunately, the OPC manufacturing is [3,4,5,6,7,8,9,10]:

- energy intensive,
- very expensive,
- a process that produces harmful greenhouse gases (GHG), such as: CO_2_, NO, NO_2_,
- an activity that degrades natural mineral deposits, such as limestone.

Therefore, it can be concluded that the production of OPC is non-ecological and has a negative impact on the natural environment, e.g., [11,12,13].

For this reason, the substitution of cement binder with other materials is recently becoming a more and more popular research topic, whereas OPC replacement materials, i.e., supplementary cementitious materials (SCMs) are increasingly used for concrete mix production, e.g., [14,15,16,17].

The main group of SCMs is industrial wastes [18,19]. Taking this into account the use of SCMs in the concrete industry reduces troublesome landfills of these materials, e.g., [20]. Furthermore, according to the literature, the most frequently used material from this collection is low calcium fly ash (LCFA) [21,22,23]:

However, according to other reports, radiologically safe [24] LCFA as a by-product of combustion in billions of power plants around the world is now becoming the basic supplement cement binder in the concrete composition, e.g., [25,26]. Moreover, substitution of OPC with LCFA causes a reduction of, i.e.:

- binder production costs,
- energy consumption,
- CO_2_ emissions.

From the reasons mentioned above this activity is certainly environmentally friendly, e.g., [27,28,29]. In general, it can be stated that the use of LCFA in the cement industry reduces both significant landfills of this waste and also reduces the use of OPC. As a result, the production of the main concrete binder is reduced, thus resulting in numerous environmental benefits, e.g., [30].

Concretes made with OPC have been used for almost 200 years, while composites with the addition of LCFA for almost 90 years. At that time, it was found that this type of materials has many beneficial characteristics, such as: increased strength after a long period of curing, increased corrosion resistance, high resistance to impact and dynamic loads, increased resistance to high temperatures, and increased electrical resistivity, e.g., [31,32,33,34,35,36,37,38]. The numerous advantages of using LCFA as concrete additives are presented, i.e., in [39,40,41]. According to the data contained therein the incorporation of LCFA: improve the workability of concrete mixture, increases the water requirement, setting time and soundness of cement paste specimens and decrease the drying shrinkage of hardened cement paste.

The curing time of the modified LCFA composites has a significant impact on improving their parameters. Due to relatively slow rate of pozzolanic reactions in LCFA concretes, in the initial period of their curing, a much less favorable effect of this binder substitute can be seen than it is the case in the subsequent curing periods of composites.

**Figure 1:**
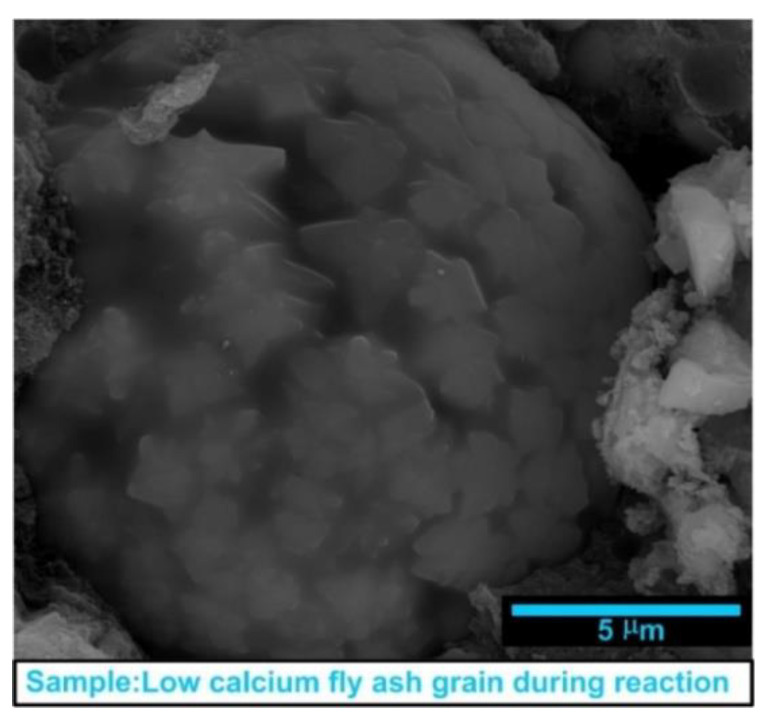
Exemplary of a fine low calcium fly ash (LCFA) grain during a pozzolanic reaction.

Furthermore, based on previous research it is concluded that the most important features of LCFA are their high reactivity and favorable morphology of the particles. It manifests itself mainly in spherical shape of grains and fine structure. Exemplary LCFA grain with such parameters, i.e., fine and well-developed particle with a diameter of 10 μm, are shown in Figure 1. The picture of LCFA grain, presented in Figure 1 and observed during Scanning Electron Microscope (SEM) studies, additionally illustrates the moment of strong pozzolanic reactions that occur in its structure.

The review of studies analyzing various parameters of composites with the addition of LCFA depending on their curing time were presented in a tabular form in the papers [42,43]. According to the information contained therein, the effects of the curing time of concretes containing LCFA have been recognized—up to a year or even longer, e.g., up to 1000 days [44]—and these are as follows:

- mechanical properties, e.g., [45,46,47,48,49],
- corrosion resistance, e.g., [44],
- chloride penetration, e.g., [50,51],
- drying shrinkage, e.g., [51],
- porosity and pore volume distribution, e.g., [46],
- heat development, e.g., [46],
- water absorption and water permeability, e.g., [48],
- hydration process, e.g., [52],
- microstructure, e.g., [45,52,53],
- crack propagation, e.g., [45],
- fracture toughness evaluated in mortar tests with LCFA, e.g., [54].

Concretes with the addition of reactive and fine-grained LCFA were also tested for fracture toughness. However, the vast majority of previous experiments assessed the parameters of fracture mechanics of composites under tension, i.e., mode I fracture, e.g., [55,56,57]. In the analyses, both linear and non-linear fracture mechanics were considered [56,57] and the obtained results showed clear qualitative convergence. In addition, generalized fracture toughness of LCFA concretes was also estimated in previous studies, e.g., [43].

However, one should not forget about the fracture toughness analysis for the 2nd and 3rd models of cracking. Especially in the case of modified concretes with a changed composition of the cement matrix, e.g., [58]. Situations, in which complex stress states determine the destruction of materials apply to both concrete composites and other brittle materials, e.g., [59,60,61,62,63,64].

Fracture toughness of young and mature concrete with the additive of LCFA, under mode III fracture, were discussed in previous papers [65,66], while, under mode II, the fracture of concretes containing the binder substitute has been evaluated so far only at a young age and in the standard period, i.e., after 28 days of curing [67]. In the case of complex stress states in concrete structures, the dominant are destructive processes in the material caused by shear as a result of the impact under mode II fracture, e.g., [68,69,70,71]. Therefore, in order to fill this gap in the literature and to obtain additional data in the evaluation of fracture toughness of concretes containing LCFA, the following article presents the test results and a comprehensive analysis of the impact of curing time (up to a year) on the development of cracks and material destruction caused by a shear force longitudinal to the edge of crack. Furthermore, the knowledge obtained in this way will have a practical application in the future in assessing the destructive processes of real structures. This is due to the fact that the special mechanical parameters of concrete, such as fracture toughness, are used in the analysis of the work of real building structures, e.g., [72].

## 2. The Importance of Research

Fracture behavior is an important issue to be taken into account in the analysis and design of concrete structure. This is due to the fact that the fracture of brittle or quasi-brittle material, such as concrete, begins at the weaker place and then, as a result of stress concentration at both ends of the crack, it spreads quickly without even increasing the load. On the other hand, the fracture toughness of concrete is strictly dependent on the input composition of the concrete mix and the materials used to make it [73]. Partial substitution of cement binder with LCFA undoubtedly changes the structure of the composite. This has a clear impact on the important parameters of the material in which characteristics will be different compared to the indicators for unmodified concrete. Therefore, this scientific problem requires in-depth research.

In structures made of unreinforced concrete there is a possibility of brittle failure due to tensile or shear stresses in the element’s sections, and microcracks in the material’s structure. Fracture mechanics deals with the problems of brittle failure, whereas the basic parameter of fracture toughness is a critical stress intensity factor—*K*_αc_ (α = I, II or III, depending on the adopted mode fracture), e.g., [74,75,76,77,78,79,80,81].

When analyzing the phenomena accompanying the destruction of materials with cement matrixes, one of the most important research problems is the connection of the structure of these materials with their strength considered in terms of fracture toughness [82,83,84,85].

Fracture toughness is an extremely important parameter determining the properties of a given material, especially a construction material. The material constants determined in compressive and tensile tests are not enough because often materials with high mechanical properties (high strength) have low fracture toughness. In this case, such materials have limited usefulness as structural materials, especially in terms of fatigue loads in a given structure [57].

Taking the above into account, the following are the issues important in the analysis of the fracture toughness of concrete composites modified with pozzolanic additives from the point of view of the microstructure of these materials. As these problems are rarely presented in the literature, they require a more detailed explanation. The fracture toughness of concrete under shear in the macroscopic approach has been presented in detail in several fundamental publications on this subject [86,87,88,89] and in the stress intensity factors handbook [90].

First of all, it should be noted that, in most construction materials the mode I fracture is the dominant case that should be taken into account in the assessment of fracture toughness [91,92]. However, in composites with cement matrices, the situation is slightly more complicated. Shear fracture toughness becomes more important in this type of material for several reasons. First of all, the fracture toughness test, according to mode II fracture, i.e., shear loading, is particularly important for composites with cement matrixes. This is due to the fact that the shearing strength of these materials is relatively low, e.g., [93]. Nogueira and Zhou et al. [94,95] even state that microcracking due to tension occurs under low to moderate load and then shear phenomena tend to dominate. Furthermore, the analysis of concrete members under shear force requires reliable material parameters [86]. On the other hand, the strength of multiphase materials, such as concrete with LCFA additive, depends on the properties of the individual phases and their percentage content in the mass of the material, as well as the mutual interaction and phenomena occurring at the phase interface.

Generally, in the case of concrete composites modified with pozzolanic additives, such as LCFA, the situation becomes even more complex and more difficult to analyze. This is due to the fact that in such materials, the development of cracks may also propagate at the boundaries of additional phases formed in the modified composite structure. As a result of the intensification of the pozzolanic reaction, some of the new products formed in concrete, such as the C-S-H phase, definitely have a positive effect on reducing the development of intra-material cracks, and thus improving the fracture toughness of the material [96]. On the other hand, the negative effect of brittle crystals of the portlandite phase (CH), which is characterized by significantly lower fracture toughness and is usually present in large amounts in unmodified concretes, was also confirmed [97].

The mechanical and strength parameters and the fracture toughness of the two main matrix phases are influenced by factors related both to their structure and the location of their occurrence in the matrix structure. The comparison of the fracture toughness test results for CH and C-S-H shows significantly worse values for portlandite compared to C-S-H. The authors of [96,97] showed that the stress intensity factor *K*_c_ is lower for the CH phase compared to C-S-H, the mixture of C-S-H/CH [96] and the cement paste [97]. The conclusions presented in [96] also reveal that in the case of C-S-H the highest fracture toughness can be obtained when CaO/SiO_2_ in this phase is 0.99. Mindess, in the conclusions presented in [98], stated that the development of cracks at the interface between aggregate and portlandite is determined by the morphology of crystals of this phase occurring around the aggregate grains.

As the CH phase is a weaker phase, with a lower fracture toughness, in relation to C-S-H, cracks in these zones of the paste most often appear along the cleavage planes of the CH phase [99]. Clusters of large portlandite crystals in the area of the aggregate contact layers, which can significantly weaken it and make it more susceptible to corrosion and brittle damage, are particularly unfavorable.

Moreover, at the boundaries of morphologically different phases, cracks are usually the result of complex stress states. Thus, it forces the analysis of the fracture toughness of such materials, which not only takes into account the first, but also the second and often the third mode fracture, e.g., [4].

Therefore, in such cases Mode II or in-plane sliding of crack faces is one of the possible fracture modes for concretes containing LCFA, which can often take place due to shear loading. Hence, civil engineers need to know the value of Mode II fracture toughness (*K*_IIc_) of modified concretes mixtures as an important design parameter [71].

It is also related to the fact that reinforced concrete elements are usually thick and often deep beams, not bars, and, in practice, the problem of brittle cracks caused by the mode II fracture may include a significant group of structures, which include, among others:

- support zones of RC beams,
- short cantilevers,
- undercut beams,
- beams loaded with point loads,
- corners of the rigid frame bridges,
- deep beams, and
- connections in precast segments.

Fracture toughness under shear of some of the above-mentioned structural elements are presented, among others, in papers [100,101,102,103,104,105,106]. The wide range of examples of constructions, in which mode II fracture may determine the destruction, show the undoubted importance of the analyzed scientific problem.

On the other hand, the fracture toughness in concretes with LCFA additive has been tested irregularly so far and it related to various types of composites. During the experiments, the first mode fracture was mainly taken into account, and the tests were usually performed after 28 days of curing. Moreover, in earlier studies, very different amounts of this industrial waste were also used. Previous papers which presented the results of the fracture toughness of concretes with LCFA addition, at Mode I fracture, are gathered in Table 1.

The author of the article also conducted research on the fracture toughness of composites modified with the additive, carried out for the mode II fracture. However, no other articles on this subject have been found in the literature. Unfortunately, the research conducted so far has been limited only to the assessment of the influence of the curing time of such materials in the first 4 weeks after preparing the batches [67]. It is commonly known, however, that the pozzolanic activity of LCFA gains intensity at a later stage from the contact of these additives with the cement paste. Therefore, it is advisable to trace the important parameter, which is the fracture toughness under mode II fracture, for a composite containing different amounts of LCFA, also after a longer period of curing.

Considering the above, this article focuses on analyzing fracture processes in LCFA concrete taking into account the mode II fracture. In general, this paper has three main goals:

- analysis of influence of the curing time on the values of critical stress intensity factor *K*_IIc_,
- evaluation of brittleness of concretes depending on the adopted material modification, and
- diagnosis of the basic pattern of crack development in specimens subjected to shear.

## 3. Experimental Section

### 3.1. Materials

As in previous studies, which evaluated fracture processes of concrete composites with the addition of LCFA [4,24,42,43,55,56,65,66,67,70], this time fracture toughness tests were also conducted with three types of concretes with different LCFA additives, i.e.:

- without the LCFA addition (LCFA-00),
- with 20% LCFA addition (LCFA-20),
- with 30% LCFA addition (LCFA-30).

Table 2 and Table 3 show: types, appearance, origin, and substantial parameters of the materials used in the studies. However, the composition of prepared concrete mixes, including water to binder ratio, is shown in Table 4.

### 3.2. Methods

In the field of experimental research, 2 technical parameters were analyzed, i.e.:

- compressive strength (*f*_cm_) and
- fracture toughness at shear (*K*_IIc_).

**Figure 2:**
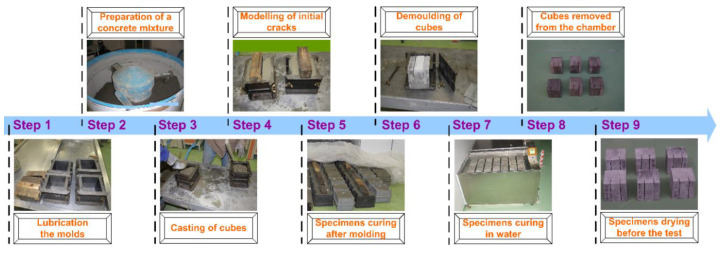
Preparation process of the specimen for fracture toughness tests.

Both strength and fracture toughness parameters were tested on 6 specimens, made for each series of concrete, after: 3, 7, 28, 90, 180, and 365 days of their curing. Cubic samples with dimensions: 150 × 150 × 150 mm were used in all studies. In the case of fracture toughness tests, the cubes contained two initial cracks, which formed during the formation of the cubes. Preparation stages of the main test specimen is shown in Figure 2.

**Figure 3:**
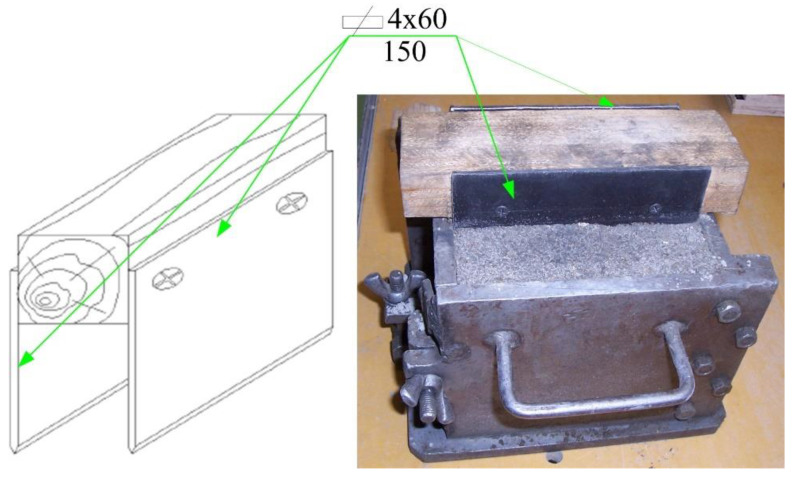
Specimen for the Mode II fracture tests during preparation (dimensions in mm).

Moreover, Figure 3 shows the process of forming initial cracks in detail, i.e., Step 4 from Figure 2. The target crack sizes were obtained by embedding in concrete cubes while they were being formed two 4 mm steel sharpened flat plates (Figure 3). Once the samples have been formed, 2 sharpened flat plates were immediately removed from the stand; see Step 6 from Figure 2. However, it should be added that the initial cracks may also be prepared mechanically, i.e., by cutting with a diamond saw. This method of the initial cracks’ preparation, in the specimens for shear fracture toughness, is shown in [111].

**Figure 4:**
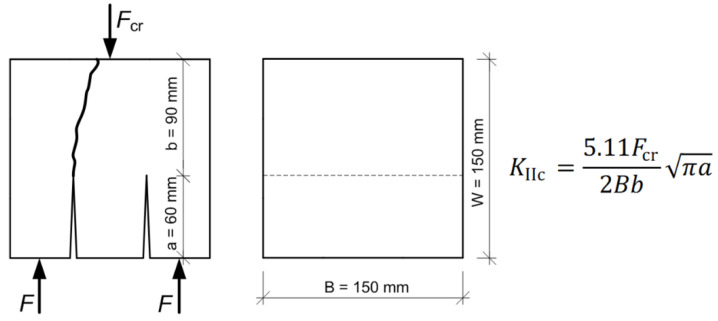
Detail of specimen used in the studies.

In addition, Figure 4 shows details for the specimen used in the fracture toughness tests, i.e.:

- its dimensions,
- loading conditions,
- designation of force *F*_cr_ causing the development of initial cracks, and
- equation for determining *K*_IIc_ parameter, according to Watkins [112].

At this point, it should be noted that the specimens used for *K*_IIc_ values were originally for cubic specimen size of 100 mm [112]. Therefore, numerical analyses were performed in order to verify the possibility of using specimens of larger sizes in such experiments, i.e., concrete cube with a side length of 150 mm. In order to achieve this goal, the new numerical model for the 3-dimensional specimen was created. We used for analyses:

- ABAQUS program, created by Dassault Systèmes Simulia Corp. from Providence, Rhode Island, USA supported by Extended Finite Element Method (XFEM) during numerical simulations,
- peak principal stress criterion for description of the crack grow.

**Figure 5:**
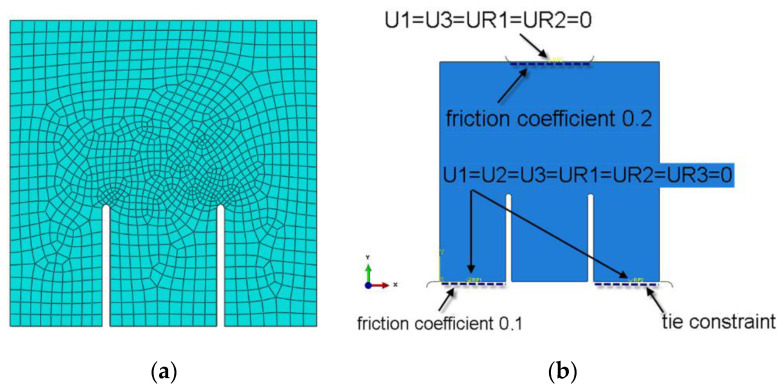
Specimen used in numerical analyses with (**a**) finite element mesh (**b**) boundary conditions: U—displacement, UR—rotation.

Moreover, Figure 5 shows finite element mesh and boundary conditions used in numerical calculations.

**Figure 6:**
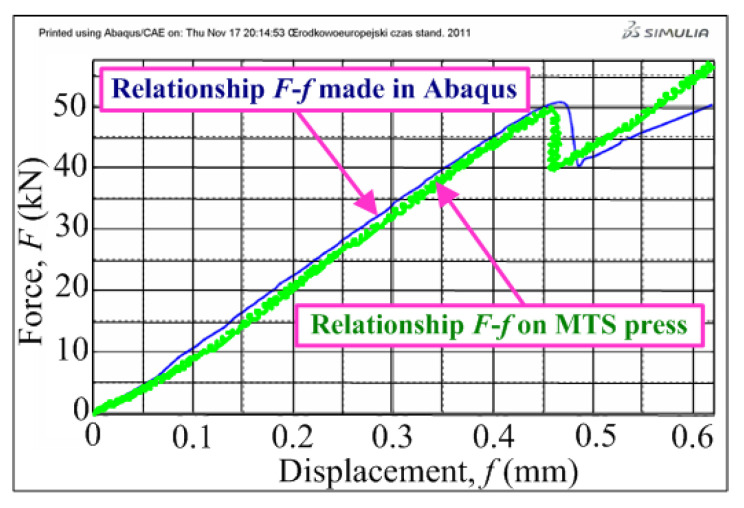
Comparison of force (*F*)–displacement (*f*) diagrams for exemplary specimen.

In the course of the numerical analyses, it was found that the numerical results were convergent qualitatively and quantitatively with the experimental results [67,113]. The convergence of the results amounted to approximately 2%, which is clearly visible by comparing the exemplary charts of load (*F*)–displacement (*f*) (Figure 6).

On this basis, it was found that it is possible to use specimens of larger sizes in the fracture toughness tests of concrete composites under Mode II fracture.

Compression strength tests were conducted using a compression machine (Walter + Bai ag, type NS19/PA1; Löhningen, Switzerland) with a maximum load of 3000 kN, whereas, during investigations of fracture toughness *K*_IIc_ press (Materials Test System, MTS; type 810; MTS Systems Corp.; Eden Prairie, MN, USA) with maximum load of 100 kN was used.

During the fracture toughness experiments, according to Mode II fracture, the following were applied:

- a displacement controlled type of tests with the MTS head velocity equal to 0.25 mm/min,
- quasi-static increase of the loading force (*F*) up to the final failure of the specimens.

**Figure 7:**
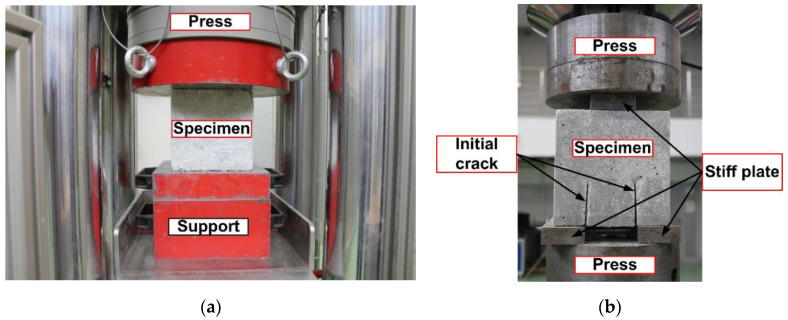
Specimens during the tests: (**a**) compressive strength; (**b**) fracture toughness.

A view of specimens on both experimental stand is shown in Figure 7.

In both studies, the specimens were loaded statically. The compressive strength tests were carried out according to the standard EN 12390-3: 2011+AC: 2012 Testing hardened concrete—Part 3: Compressive strength of test specimens [114]. However, during the fracture toughness tests, the following were evaluated:

- load (*F*)–displacement (*f*) relationship and
- fracture toughness *K*_IIc_ parameter.

Summing up all the assumptions necessary for the implementation of experimental tests and scientific goals defined in the article, they are clearly summarized in Table 5. This table outlines the experimental program and reports the shape, as well as the geometric dimensions of the concrete specimens. Macroscopic examinations were carried out in 6 time periods. Particularly, the time evolution of all investigations has been monitored by testing 6 specimens for each type of concrete (Table 5). Therefore, a total of 216 specimens were tested.

## 4. Results and Discussions

### 4.1. Analysis of Changes in Concretes Mechanical Parameters Depending on Ages

**Figure 8:**
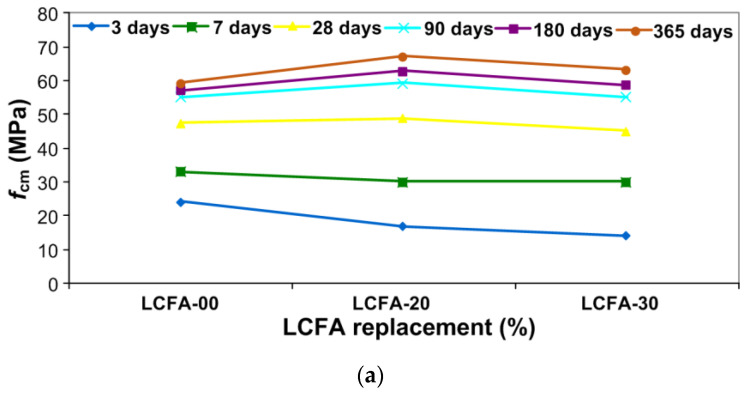

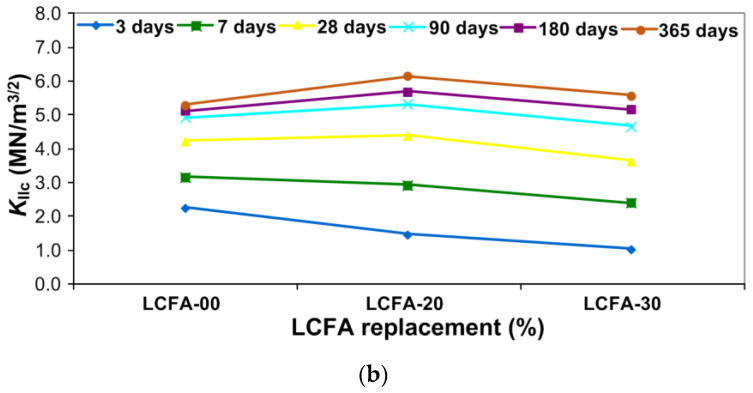
Mechanical parameters of analyzed concretes samples depending on ages: (**a**) compressive strength; (**b**) fracture toughness at shear.

Figure 8 shows the distributions of analyzed mechanical parameters from the 3rd to 365th day of specimens curing. Moreover, Table 6 summarizes the average results of the two analyzed parameters with the coefficients of variations—ν.

**Figure 9:**
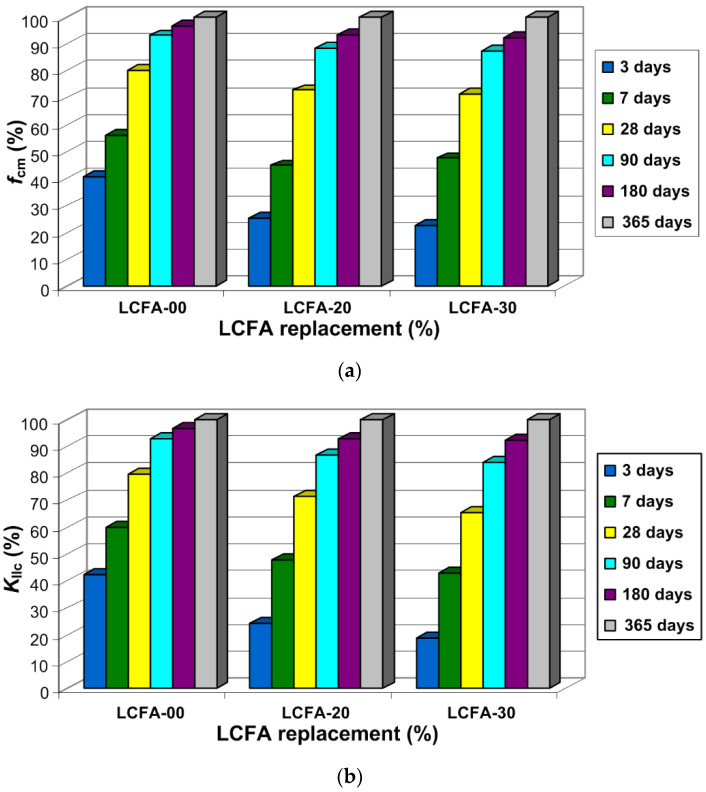
Relative changes of the analyzed parameters over time: (**a**) compressive strength; (**b**) fracture toughness at shear.

The comparison of dependencies from both experiments indicates a clear similarity between the two analyzed mechanical parameters, in which changes in time took place in a very similar way. The main observations arising from the analysis of the graphs from Figure 8 and Figure 9 and Table 6 are as follows:

- the applied LCFA additive reduces the early fracture toughness of cement concrete, as well as compressive strength of composite, i.e., in the first week of curing;
- 1 month old concretes with addition of 20% LCFA are characterized by higher *K*_IIc_ and *f*_cm_ parameters than the reference concrete sample;
- during the standard period, i.e., after 28 days and longer periods of curing LCFA-20 had the highest strength and fracture toughness, which was influenced by the intensification of the pozzolanic reaction after a longer time of curing (Figure 1);
- the LCFA-30 composite only after 3 months achieved parameters similar to those obtained for LCFA-00 and LCFA-20 concretes samples;
- concrete with more LCFA will achieve higher *K*_IIc_ and *f*_cm_ values after half a year compared to the reference concrete, while they were still lower than the results obtained for LCFA-20 (even after one year); and
- the greatest increases in *K*_IIc_ and *f*_cm_ were observed: in the case of reference concrete during the first 28 days, in the case of concretes containing LCFA in the time period above 4 weeks.
- the greatest dispersion of results, represented by the highest values of ν, was observed in the concrete with the shortest curing time (Table 6).

Additionally, in order to more accurately illustrate the increases in the analyzed mechanical parameters in particular time periods, Figure 9 presents the relative changes in *f*_cm_ (Figure 9a) and *K*_IIc_ (Figure 9b) of the analyzed composites.

On the basis of the above charts, an in-depth analysis of the obtained test results was carried out, which is presented below. The following were observed:

- significantly higher values of both analyzed mechanical parameters in the reference concrete samples compared to concrete samples modified by LCFA in the first of the analyzed curing periods., i.e., after 3 days; during this period, reference concrete had more than 40% of the annual compressive strength, whereas, in concretes with LCFA additives, this strength did not even reach 30% of the final strength (Figure 9a), whereas fracture toughness was lower by 35% and 54% for LCFA-20 and LCFA-30, respectively, in comparison to the value obtained for LCFA-00 (Figure 9b);
- a clearer dynamics of strength and fracture toughness increase in composites with LCFA after a week; nonetheless, during this period, concrete without the LCFA additive was still characterized by a higher relative strength and fracture toughness (Figure 9);
- sharp increase of *f*_cm_ and *K*_IIc_ for concretes with the LCFA additive between the 7th and 90th day of curing (Figure 9); during this time, the values of analyzed mechanical parameters for these materials increased more than 80% for LCFA-20 and above 90% for LCFA-30; and
- stable and small increase in *f*_cm_ and *K*_IIc_ in all series of concretes after 180 and 365 days of curing; during these periods, the most significant changes were observed in concrete with a greater amount of pozzolanic additive, i.e., LCFA-30 (Figure 9).

Table 7 summarizes the results of own tests of fracture toughness of concrete, after 28 days of curing, with the results obtained by other researchers carried out on samples of the same dimensions. According to the data contained therein, concrete with addition of 20% LCFA, made on gravel aggregate, was characterized by high fracture toughness—*K*_IIc_ = 4.39 MN/m^3/2^, similar to the results obtained for concretes made on broken aggregates with very favorable parameters, i.e.: basalt—*K*_IIc_ = 4.45 MN/m^3/2^, limestone—*K*_IIc_ = 4.64 MN/m^3/2^ and granite—*K*_IIc_ = 5.14 MN/m^3/2^. On the other hand, the concrete of the LCFA-30 series had a very low fracture toughness compared to previous test results—*K*_IIc_ = 3.65 MN/m^3/2^.

### 4.2. Assessment of Material Fracture Processes at Mode II Fracture

**Figure 10:**
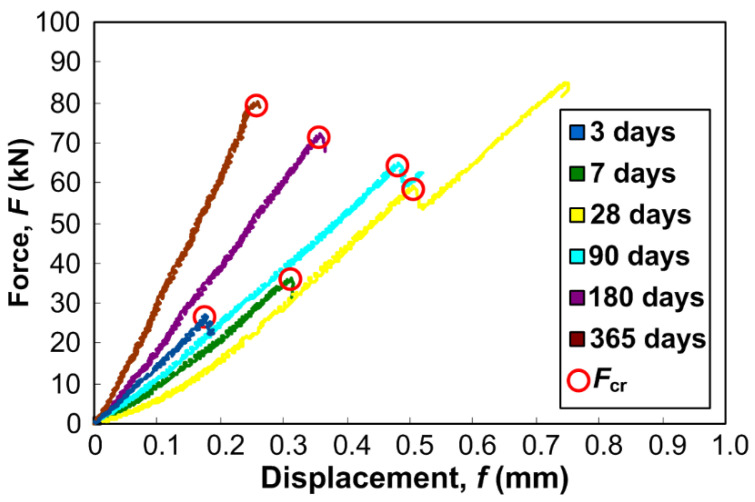
Exemplary force–displacement diagrams for LCFA-20 specimens.

Figure 10 shows examples of graphs of function *F*–*f* prepared for some of the specimens of concrete LCFA-20, in the analyzed periods of time. Additionally, points in which forces *F*_cr_ occurred necessary to determine the parameter *K*_IIc_ (Figure 4) are marked on destruction curves.

When analyzing the graphs *F*–*f* obtained for all tested concretes in the time interval between the 3rd and 365th day, two different cases, describing the shape of the curves and the time in which critical forces were reached, can be observed. Concrete samples with LCFA additives are mainly characterized by a quasi-static process of failure. Initial cracks in these composites were most often developed in two stages, i.e., crack developed with a clear division into: the moment of the initial damage at the force *F*_cr_ and final stage of crack propagation up to the final failure. This case can be clearly seen in Figure 7 in relation to 28-day concrete. Concrete samples without the LCFA additives were characterized by a brittle fracture. In such situations, cracks initially developed steadily, after this a sharp drop on the graph *F*–*f* and clear development of cracks were observed. The second type of failure was also characteristic for concrete samples LCFA-20 after: 3, 180, and 365 days of curing (Figure 10).

In the case of composites that had a high fracture toughness, the values of critical forces reached 80 kN, whereas, for materials with low *K*_IIc_, they ranged from 10 to 20 kN. For concretes with the highest values of critical stress intensity factors, i.e., LCFA-20/365, critical forces above 80 kN were noticed, whereas, for a number of specimens from LCFA-30/3, forces *F*_cr_ they did not even exceed 10 kN.

At this point, it should be added that the *F*–*f* diagrams for two other analyzed concrete specimens, i.e., LCFA-00 and LCFA-30—apart from the differences related to their brittleness, described above—were similar qualitatively to the curves shown in Figure 10. Apart from the fact that the graphs for the reference concrete showed a brittle failure mode in almost each of the analyzed periods of curing, in contrast to concrete specimens containing LCFA, the main observed difference between them were the different values of the *F*_cr_ forces that were observed during the experiments.

However, an in-depth evaluation of the macroscopic cracks formed in the specimens after the tests was conducted in order to more precisely illustrate the presented observations. For this purpose, diagnostics with the use of up-to-date and precise digital image correlation (DIC) technique were used [55,56,57].

**Figure 11:**
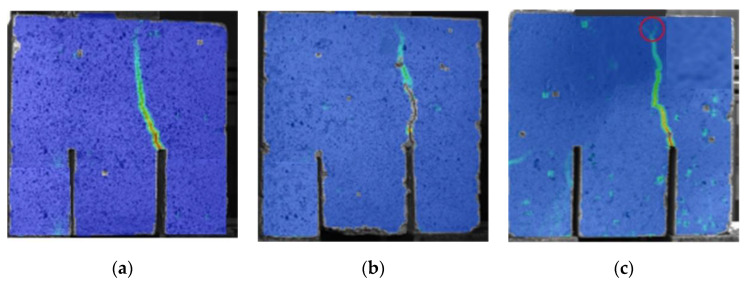
Examples of crack shapes observed in the studies using digital image correlation (DIC) technique: (**a**) quasi-straight crack for concrete specimen LCFA-00; (**b**) slightly curved crack for concrete specimen LCFA-20; (**c**) strongly curved crack for concrete specimen LCFA-30.

The example of crack shapes observed in the studies using DIC technique in the Mode II fracture for concretes after 28 days of their curing were presented in Figure 11. This figure shows:

- quasi-straight crack, occurring mainly in the most brittle concrete, i.e., LCFA-00-00 (Figure 11a),
- slightly curved crack, which is related with the most common LCFA-20 concrete (Figure 11b), and
- clearly and strongly curved crack with small branches (visible in the final stage of crack propagation and marked with a red line) which was observed mainly in the LCFA-30 concrete (Figure 11c).

**Figure 12:**
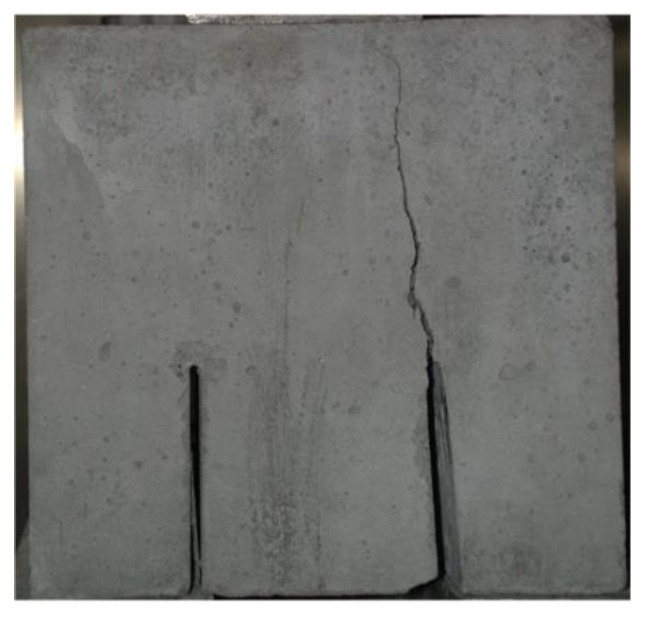
Characteristic crack path observed in the tests.

When analyzing the processes of cracking of cubes, it should be mentioned that the most of the specimens were destroyed as a result of typical shearing of concrete. A macroscopic crack, causing destruction of the material, appeared at the top of the modeled initial crack and then developed upwards to the place of force transfer through the steel plate. An example of a sample with a crack of this type is shown in Figure 12.

**Figure 13:**
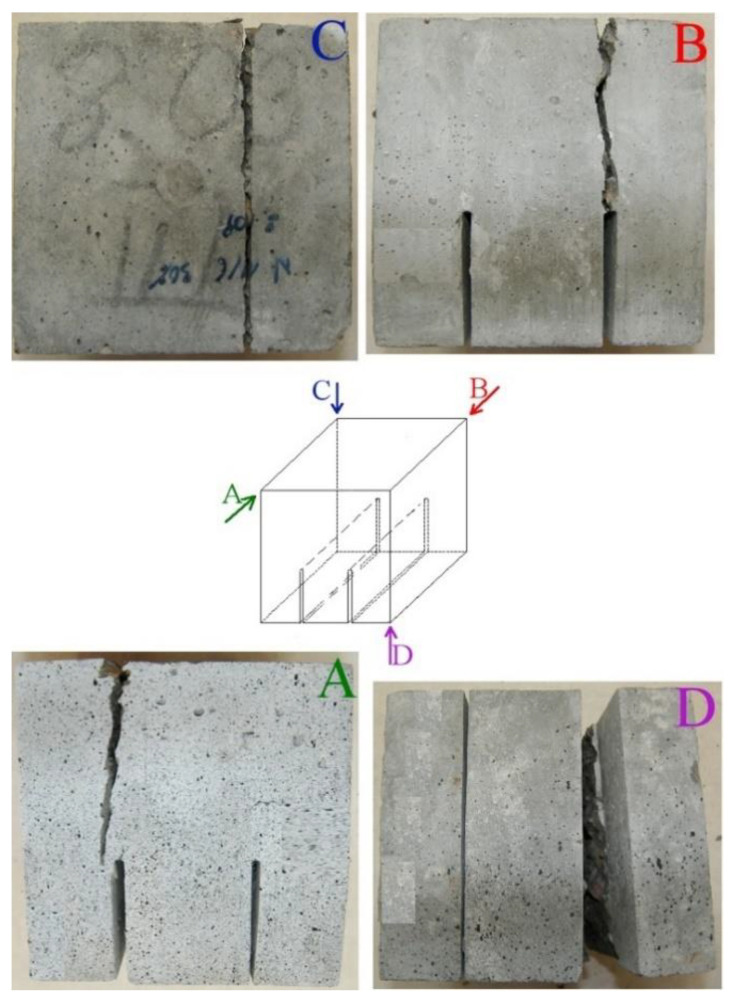
View of cracks occurring on the characteristic planes of the specimen: A–D—description in the text.

At this point, it is worth noting that the above failure pattern is associated with the occurrence of compressive and tensile stress trajectories arising in the loaded element. On the other hand, the shear of the sample is along a broken line resulting from the action of variable directions of principal stresses. This phenomenon has been confirmed on the basis of elastoplastic tests carried out many years ago. As proof of this, Figure 13 presents an in-depth analysis of the shearing process of a compact shear sample. For this purpose, pictures of the crack visible from various angles were taken for the specimen cube during its destruction. Figure 13 shows the same crack after the destruction of the specimen, which is visible on 4 significant planes, i.e., from the bottom (Figure 13D), from the top (Figure 13C), and both parts of the cube on which the initial cracks were modeled (Figure 13A,B). The pictures clearly show an almost perfect shearing of the cube. It was caused by evident separation of one side of the specimen after the destruction by creating a large crack along the entire section height above the initial crack (Figure 13).

### 4.3. Evaluation of the Microstructure of Analyzed Concretes

The conclusion about the effectiveness of the used concrete modifier should be based on the results of the tests of mechanical parameters and the assessment of the structure of these materials. It is important as the changes in the composite phase system later influence its behavior under load, the manner of crack propagation, and, finally, the material’s fracture toughness; see Section 2. LCFA are pozzolanic additives. Their percentage, in the general composition of the binder, affects the rate of reactions occurring during the formation of the composite structure and the proportions of the main phases in the cement matrix.

Qualitative changes in the concrete structure are best visible in the scanning electron microscope (SEM) images. Quantitative relationships between the individual components of the matrix can be identified additionally using an Energy Dispersive Spectroscopy (EDS) spectral analyses. A good indicator of the progress of the pozzolanic reaction and the degree of reaction of LCFA is the assessment of changes in CaO/SiO_2_ ratio in the locations of C-S-H phase, e.g., [117,118]. Therefore, the presented structural studies focused on the assessment of the phases, visible in particular composites, and CaO/SiO_2_ ratios. In each of the concretes, the locations with clearly distinct C-S-H, CH phases, and ettringite (E) phases were analyzed.

**Figure 14:**
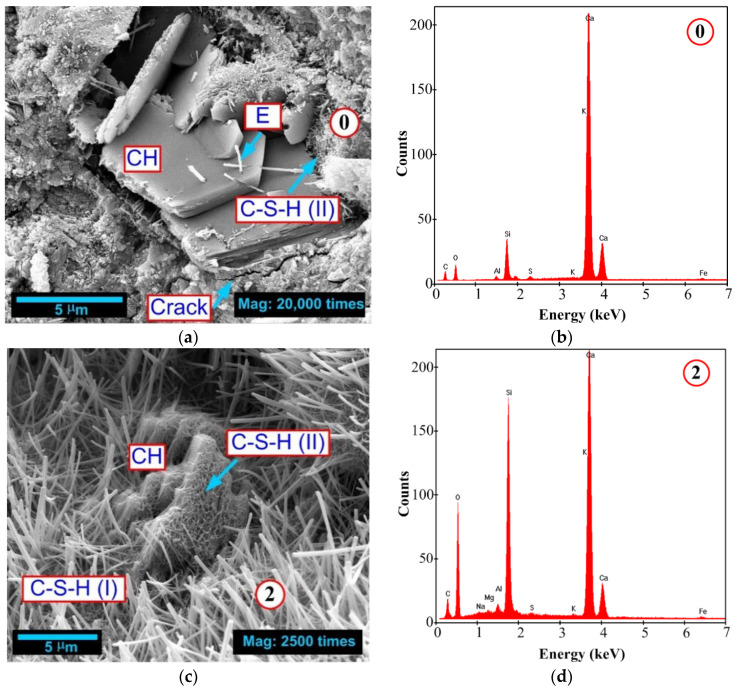

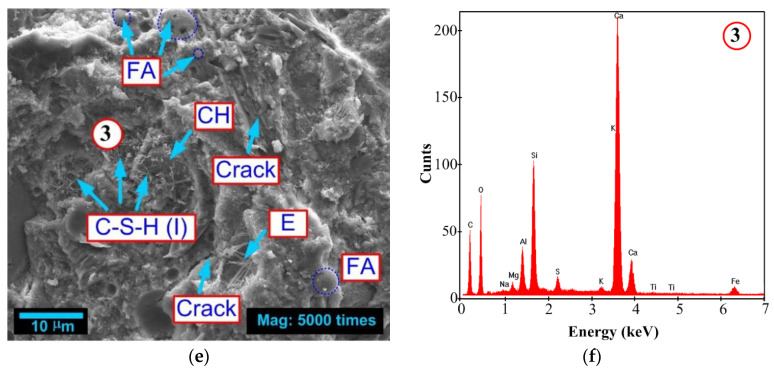
Microstructure of studied concrete specimens with EDS analyses after 28 days of curing: (**a**,**b**) LCFA-00; (**c**,**d**) LCFA-20; (**e**,**f**) LCFA-30.

The microstructural testing was carried out using a QUANTA FEG 250 (FEI Company; Hillsboro, OR, USA). SEM pictures with EDS—EDAX analyses, at characteristic points (marked with numbers 0, 2, and 3) for each of the concretes are shown in Figure 14. Furthermore, this figure shows the locations of the main phases in the concrete that affect its mechanical parameters. Samples for microstructural tests were taken from previously damaged cubes, after the fracture toughness tests, according to mode II fracture. 28-day concrete samples were thoroughly assessed.

The analysis of the phases occurring at the concrete interfaces showed that the composite with 20% LCFA additive had the most developed structure and these were mainly fibrous C-S-H (I) and honeycomb phases, i.e., C-S-H (II). Characteristically, the C-S-H phases in this material were so extensive that the reaction of CH crystals was clearly visible. This may indicate the reactions of transformation of the less favorable portlandite phase into the favorable C-S-H phase occurring in this concrete (Figure 14c).

In the reference concrete LCFA-00, clusters of ettringite needles, occurring in the pores of the matrix and portlandite crystals, were visible. There were also small amounts of C-S-H (II) phase, but the main phase occurring in these composites were large hexagonal CH plates with overgrown ettringite needles (Figure 14a).

In the concrete of LCFA-30 series, the unreacted grains LCFA with a diameter of a few micrometers were clearly visible (their position is shown by the blue dashed lines), while the structure of this concrete contained various types of slightly intense phases (Figure 14e).

Additionally, the occurrence of microcracks inside the concrete structure of the LCFA-00 and LCFA-30 series was also observed. In the first material, the damage was in a characteristic location, i.e., on the border between the brittle plates of portlandite and the compact C-S-H phase (Figure 14a). In addition, in the concrete LCFA-30, the microcracks also ran in the CH crystal structure (Figure 14e).

The conducted EDS analyses, from the Figure 14, clearly confirmed the differences in the amount of C-S-H in particular composites. The changes in the obtained spectra can be attributed to the variable content of LCFA. First of all, the EDS analysis confirms that the molar ratio CaO/SiO_2_—phase C-S-H formed as a result of the pozzolanic reaction in concretes containing LCFA additive—is much lower than the same molar ratio of C-S-H in concrete LCFA-00 (Figure 14b,d,f). The change in CaO/SiO_2_ for C-S-H is clearly visible when comparing the reference concrete and the concrete with the addition of 20% LCFA. For LCFA-00, the ratio CaO/SiO_2_ is 7, while, for concrete, LCFA-20 is only approximately 1.2 (Figure 14b,d). In concrete LCFA-30, the value of CaO/SiO_2_ is approximately 2.0 and is also lower than in LCFA-00, but slightly higher than in LCFA-20 (Figure 14f).

The differences in the ratios CaO/SiO_2_ in particular concretes, containing LCFA, are related to the different intensity of the pozzolanic reaction of these materials, which was observed on the exemplary LCFA grain; see Figure 1. This is confirmed by SEM images and tests of mechanical parameters of concrete specimens. A lower value of the molar ratio CaO/SiO_2_ indicates that most of the LCFA additive reacted with CH, e.g., [117]. This causes a significant increase in the C-S-H phase and, consequently,

- more compact material structure (Figure 14),
- higher compressive strength of concrete (Figure 8a), and
- higher macroscopic fracture toughness of the composite (Figure 8b).

The presented results of experimental research may be helpful for concrete mixture designers and technologists dealing with the selection of proportions of concrete components in terms of their use in specific concrete and reinforced concrete structures. The results obtained on the basis of the conducted extensive macroscopic and microstructural studies will be useful mainly in the selection of the composition of modified concrete composites LCFA. The detailed conclusions resulting from the content of the article, which are presented below, may enable a more conscious use of the industrial waste in structures, in which shear is the dominant force.

## 5. Conclusions

Based on the obtained test results, the following conclusions can be drawn:

1. The LCFA as an active additive in the amount of 20% and 30% causes changes in the values of fracture toughness at shear and compressive strength of concrete composites.
2. The analyzed parameters of *K*_IIc_ and *f*_cm_ depend on the curing time of individual concrete sample.
3. At early age the maximum fracture toughness occurs in reference concrete. It is 2.26 MN/m^3/2^ at 3 days, and 3.18 MN/m^3/2^ at 7 days.
4. Twenty percent LCFA additive ensures high fracture toughness in mature concrete. It is 4.39 MN/m^3/2^ at 28 days, 5.33 MN/m^3/2^ at 90 days, 5.70 MN/m^3/2^ at 180 days, and 6.14 MN/m^3/2^ at 365 days.
5. Concrete with 30% LCFA additive are characterized by high fracture toughness only after 180 days of curing. It is 5.16 MN/m^3/2^ at 180 days, and 5.58 MN/m^3/2^ at 365 days.
6. The process of crack propagation in reference specimens is brittle with straight crack, while, in specimens with the LCFA additive, quasi-plastic with curved cracks (Figure 8 and Figure 9).
7. Specimens of compact shear type, during tests according to mode II fracture, underwent shear at the extension of the initial crack (Figure 12 and Figure 13).
8. The microstructure of mature concrete specimens is characterized by: the content of the portlandite phase in the reference concrete with fractures at the phase boundaries and a high content of the C-S-H phase of types (I) and (II), as well as the visible transformation processes of the phase CH in the phase C-S-H in the concrete LCFA-20, weakly developed phases, and unreacted LCFA grains and microcracks in concrete with 30% LCFA additive (Figure 14).
9. Twenty percent LCFA additive causes a strong pozzolanic reaction in the cement matrix, thanks to which the molar ratio in this material is reduced to the level of 1.2, while, in concrete with a higher content of the additive, it is 2.0, and in non-modified concrete at 7.0.
10. The presented test results may be helpful in selecting the composition of concrete mixtures containing LCFA to be used in concrete and reinforced concrete structures subjected to shear loads.

## Figures and Tables

**Table 1:** Papers which presented test results of the fracture toughness of concretes containing LCFA during Mode I loading.

Type of Tested Concrete	Addition of LCFA (%)	Reference
Plain	0, 25, 45	[107]
Plain	0, 10, 20, 30	[57]
Plain	0, 20, 30	[55,56]
High Performance	0, 25	[108]
High Performance	0, 10, 40	[109]
Lightweight High Strength	As coarse aggregate	[110]

**Table 2:** Properties of binders used in this study.

Material	Class	Specific Surface (m^2^/g)	Specific Gravity (g/cm^3^)	Acquisition Place
OPC 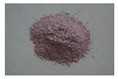	32.5 R *	0.33	3.11	Chełm Cement Plant, Chełm, Poland
LCFA 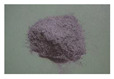	F **	0.36	2.14	Puławy Power Plant, Puławy, Poland

* cement with rapid strength and short curing time, ** siliceous fly ash.

**Table 3:** Properties of aggregates used in this study.

Material	Type and Size	Specific Density (g/cm^3^)	Compressive Strength (MPa)	Deposit Occurrence
Fine aggregate 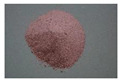	Pit sand 0–2 mm	2.60	33	Markuszów deposit, Markuszów, Poland
Coarse aggregate 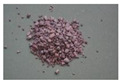	Gravel 2–8 mm	2.65	34	Las Suwalski deposit, Las Suwalski, Poland

**Table 4:** Concrete mix design (kg/m^3^).

Concrete Series	OPC	LCFA	%LCFA	Sand	Gravel	Water	Water/Binder	Plasticizer
LCFA-00	352	0	0					
LCFA-20	282	70	20	676	1205	141	0.4	2
LCFA-30	246	106	30					

**Table 5:** Experimental program.

Type of Test	Geometry of Specimens (mm)	Load Diagram	Curing Time of Specimens (days)	Number of Specimens
Compression	150 × 150 × 150	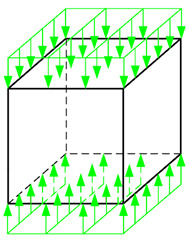	3, 7, 28, 90, 180, 365	6 in all periods
Fracture toughness	150 × 150 × 150	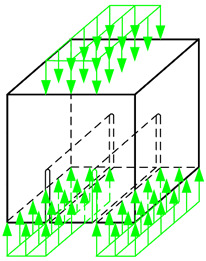	3, 7, 28, 90, 180, 365	6 in all periods

**Table 6:** The results of analyzed mechanical parameters.

Concrete Series	Age (Days)	*f*_cm_ (MPa)	ν(*f*_cm_) (%)	*K*_IIc_ (MN/m^3/2^)	ν(*K*_IIc_) (%)
LCFA-00	3	24.23	10.73	2.26	16.81
	7	33.18	7.74	3.18	12.89
	28	47.51	4.58	4.24	9.43
	90	55.13	4.55	4.93	7.10
	180	57.22	4.33	5.12	5.86
	365	59.25	4.15	5.31	4.52
LCFA-20	3	16.95	17.99	1.48	22.97
	7	30.12	10.06	2.93	13.65
	28	48.96	6.17	4.39	11.62
	90	59.35	4.72	5.33	7.69
	180	62.81	4.01	5.70	5.61
	365	67.29	3.49	6.14	4.72
LCFA-30	3	14.23	25.23	1.05	23.81
	7	30.06	11.88	2.40	12.92
	28	45.10	7.87	3.65	11.51
	90	55.11	5.63	4.68	8.12
	180	58.83	4.86	5.16	6.59
	365	63.27	3.95	5.58	4.84

**Table 7:** Comparison of the results of own tests of the fracture toughness *K*_IIc_ with the results of other researchers.

Type of Tested Concrete	*K*_IIc_ (MN/m^3/2^)	Reference
LCFA-00	4.24	Own tests
LCFA-20	4.39	Own tests
LCFA-30	3.65	Own tests
Basalt concrete	4.45	[58,115,116]
Limestone concrete	4.64	[58,116]
Granite concrete	5.14	[58,115,116]
Burned shale concrete	3.54	[58,115,116]
